# Parent-Targeted Mobile Phone Intervention to Increase Physical Activity in Sedentary Children: Randomized Pilot Trial

**DOI:** 10.2196/mhealth.3420

**Published:** 2014-11-10

**Authors:** Robert L Newton Jr, Arwen M Marker, H Raymond Allen, Ryan Machtmes, Hongmei Han, William D Johnson, John M Schuna Jr, Stephanie T Broyles, Catrine Tudor-Locke, Timothy S Church

**Affiliations:** ^1^Pennington Biomedical Research CenterPopulation and Public HealthBaton Rouge, LAUnited States; ^2^Ohio UniversityPatton College of EducationAthens, OHUnited States; ^3^Pharmaceutical Product DevelopmentBiostats and ProgrammingAustin, TXUnited States; ^4^Pennington Biomedical Research CenterBiostatisticsBaton Rouge, LAUnited States; ^5^Oregon State UniversityExercise and Sport Science ProgramSchool of Biological and Population Health SciencesCorvallis, ORUnited States

**Keywords:** mobile health, physical activity intervention, child, parents, pedometers, text messaging

## Abstract

**Background:**

Low levels of moderate-to-vigorous physical activity are associated with adverse health consequences.

**Objective:**

The intent of the study was to determine the feasibility and efficacy of a 12-week physical activity promotion program targeting children, which was delivered to parents through mobile phones.

**Methods:**

Potential participants were recruited through advertisements placed in the newspaper, local hospitals and schools, and an email listserv. Sedentary children aged 6-10 years were randomly assigned to a minimal (MIG) or intensive (IIG) intervention group. Parents in the MIG were given a goal to increase (within 1 month) and maintain their child’s activity at 6000 pedometer steps/day above their baseline levels and to monitor their child’s steps daily. Parents in the IIG were given the same steps/day and monitoring goals, in addition to text messages and articles containing additional behavioral strategies (based on the Social Cognitive Theory) designed to promote their child’s physical activity. The intervention components were delivered via mobile phone. Anthropometrics, body composition, and questionnaires were administered in a clinic. Children wore a New Lifestyles pedometer (NL-1000) each day throughout the intervention and parents were to monitor their child’s step counts daily.

**Results:**

Out of 59 children who screened for the study, a total of 27 children (mean age 8.7, SD 1.4 years; 56%, 15/27 female; 59%, 16/27 African American) were enrolled and completed the study. Overall, 97.90% (2220/2268; 98.20%, 1072/1092 for MIG; 97.60%, 1148/1176 for IIG) of expected step data were successfully entered by the parent or study coordinator. Parents in the MIG and IIG were sent approximately 7 and 13 text messages per week, respectively, averaged over the course of the study. IIG parents accessed an average of 6.1 (SD 4.4) articles over the course of the intervention and accessed a fewer number of articles in the last month compared to the first 2 months of the study (*P*=.002). Children in both the MIG and IIG significantly increased their physical activity, averaged over 12 weeks, by 1427.6 (SD 583.0; *P*=.02) and 2832.8 (SD 604.9; *P*<.001) steps/day above baseline, respectively. The between group difference was not statistically significant (*P*=.10; effect size=.40), nor was the group by time interaction (*P*=.57). Regardless of group assignment, children who significantly increased their physical activity reported greater increases in physical activity enjoyment (*P*=.003). The number of behavioral articles accessed by IIG parents was significantly correlated with change in children’s steps/day (*r*=.575, *P*=.04). Changes in children’s steps/day were unrelated to changes in their body composition, mood, and food intake.

**Conclusions:**

Parent-targeted mobile phone interventions are feasible, yet more intense interventions may be needed to support parents’ efforts to increase their children’s physical activity to levels that approximate national recommendations.

**Trial Registration:**

Clinicaltrials.gov NCT01551108; http://clinicaltrials.gov/show/NCT01551108 (Archived by WebCite at http://www.webcitation.org/6TNEOzXNX).

## Introduction

Low levels of moderate-to-vigorous physical activity are associated with adverse health consequences. Specifically, epidemiological studies demonstrate that low levels of physical activity are positively associated with childhood obesity [1] . Accumulating evidence suggests that total physical activity levels and time spent in moderate-to-vigorous physical activity are inversely associated with cardiovascular disease (CVD) and diabetes risk factors [2-9]. Therefore, increasing intensity of physical activities or time spent being physically active may have a significant impact on reversing excessive adiposity in children and reducing their risk of developing chronic disease.

Parents have an important role in teaching and encouraging their children to be physically active. For example, cross-sectional and prospective studies provide evidence that parental support and rules, as well as physical activity modeling and co-participation, are positively associated with objectively measured moderate-to-vigorous physical activity levels in children [10]. In addition, favorable parental perceptions of neighborhood safety and reports of frequent family trips to the park are related to parental reports of children’s increased time in free play [11]. Despite these correlational findings, reviews of interventions for children in which physical activity promotion was the main component, or at least one of the intervention components, concluded that family-based interventions have not yet demonstrated strong evidence of effectiveness [12-15]. However, the authors of the reviews noted that many of the family-based interventions have had methodological limitations, including failure to use randomized comparative interventions, high dropout rates, and/or a reliance on self-reported outcome assessments [12-15]. These limitations, coupled with the fact that family-based approaches have shown great success with weight management in children [16-18], suggest that there is a need to improve upon the methods used in family-based interventions targeting physical activity promotion in children.

There has been an increase in the use of mobile phones as an intervention delivery strategy. Mobile phones contain several features appealing to researchers. Mobile phones are portable, which provides the opportunity to collect real-time data [19] and to aid in self-monitoring [20]. Furthermore, participants view text messages as more convenient and effective than other types of communication [21], text messages can be personalized and can augment behavior change strategies [22], and mobile phone-based interventions have been shown to be cost-effective compared to alternative interventions [23]. Several different mobile phone-based interventions have been designed to increase physical activity in children. These interventions were either solely focused on increasing physical activity [24] or offered a physical activity component as part of a larger intervention (eg, weight loss, diabetes management) [25-29]. Importantly, reviews [30-32] have concluded that mobile health interventions can increase physical activity in children, though few mobile phone-based interventions have been conducted. To date, none of these mobile phone studies have delivered the physical activity intervention exclusively to parents. Therefore, interventions delivered through mobile phones promise a novel approach to family-based physical activity promotion.

Few family-based interventions have exclusively targeted children’s physical activity [33-37]. These studies have not consistently produced positive results. Mobile phone-based interventions are promising, yet to the best of our knowledge, no childhood physical activity study has delivered a mobile phone-based intervention exclusively to the child’s parents with the intent of increasing the child’s physical activity. In addition, the clustering of risk factors for CVD develops between 6 and 9 years of age [38], providing an appropriate target age-range for intervention. Therefore, the primary aim of this pilot study, “P-Mobile”, (trial registration NCT01551108) was to determine the feasibility of delivering a physical activity promotion program targeting 6-10 year old children that is delivered to parents through mobile phones. A second aim was to determine whether or not the intervention could elicit objectively determined increases in children’s physical activity.

## Methods

### Participants

Children who were 6 to 10 years of age, physically capable of exercise, and sedentary were eligible for the study. A parent or legal guardian of each participating child was eligible if they owned a mobile phone with Internet access and text message capabilities. Children were excluded if they were diagnosed with a serious medical disorder (eg, cancer within the last five years, cardiovascular disease). Families were compensated US $200 for their time, mobile phone data use, and travel costs. All study procedures were approved by the Pennington Biomedical Research Center Institutional Review Board.

### Procedures

**Figure 1:**
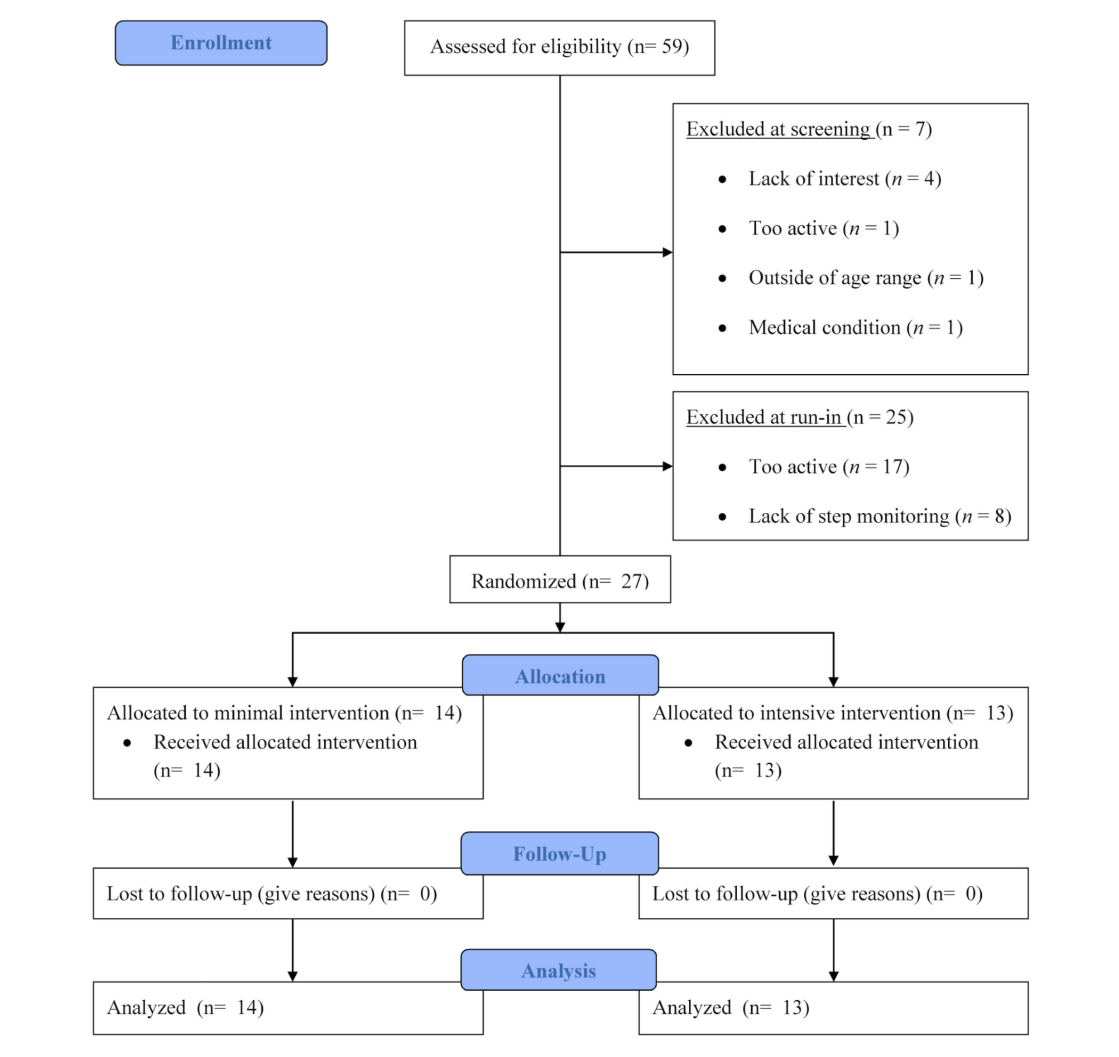
Study flowchart.

Potential participants were recruited through advertisements placed in the newspaper, posted in local hospitals and schools, and delivered through a Pennington Biomedical Research Center email listserv targeting registered individuals interested in participating in research. Once self-identified, one parent completed an initial telephone screen to determine eligibility for themselves and their child. If the parent-child dyad was eligible following the phone screen, they attended a clinic screening visit at the Pennington Biomedical Research Center (Louisiana). The dyad was oriented to the study and then written informed consent was obtained from the parent and written assent was obtained from the targeted child. The baseline assessment (see Measures below) was then conducted. At the end of the clinic visit, the targeted child was fitted with a pedometer (New Lifestyles 1000/NL-1000), the parent was required to use their mobile phone to respond to a text message sent from the study coordinator, and the parent had to access the study website. The dyad was sent home with the following instructions: the child was to engage in their normal level of activity and the parent was instructed to use their mobile phone to access the study website [39] to record their child’s step count each night after the child laid down to go to bed. This website was formatted for a mobile phone and contained a webpage to enter the date and the child’s step count. Following the clinic visit, the dyad was sent home to begin the 7-day run-in period the following morning. The run-in period was designed to assess the targeted child’s baseline physical activity levels and the parent’s compliance with monitoring the child’s step counts. The dyad was eligible for the study if girls averaged <9500 steps/day or boys averaged <12,500 steps/day (sex-specific cut points indicative of sedentary behavior in children) [40] and parents entered at least 5 days of step counts into the study website across the 7-day run-in period (evidence of ability to comply with data recording requirements). The dyad was not made aware of these eligibility criteria so that they did not alter their behavior in order to qualify for the study. Those dyads meeting eligibility criteria at the end of the run-in period were randomly assigned to either the minimal (MIG) or intensive (IIG) intervention group. Among the 59 dyads showing interest, there were 27 dyads eligible for the study, 14 were randomized into the MIG and 13 into IIG (Figure 1).

### Interventions

#### Overview

This study was a randomized comparative behavioral trial of a minimal versus an intensive intervention delivered to parents via mobile phone with the purpose of increasing physical activity in sedentary children. A block randomization procedure was generated by a study statistician (HH) utilizing SAS software, with a block size of four. The randomization sequence was placed in sealed, numbered envelopes. The clinic coordinator opened the next envelope in the sequence after a participant successfully completed all eligibility criteria. Children in both study groups were instructed to wear a study-provided pedometer every day during the course of the 12-week intervention.

#### Minimal Intervention Group

**Figure 2:**
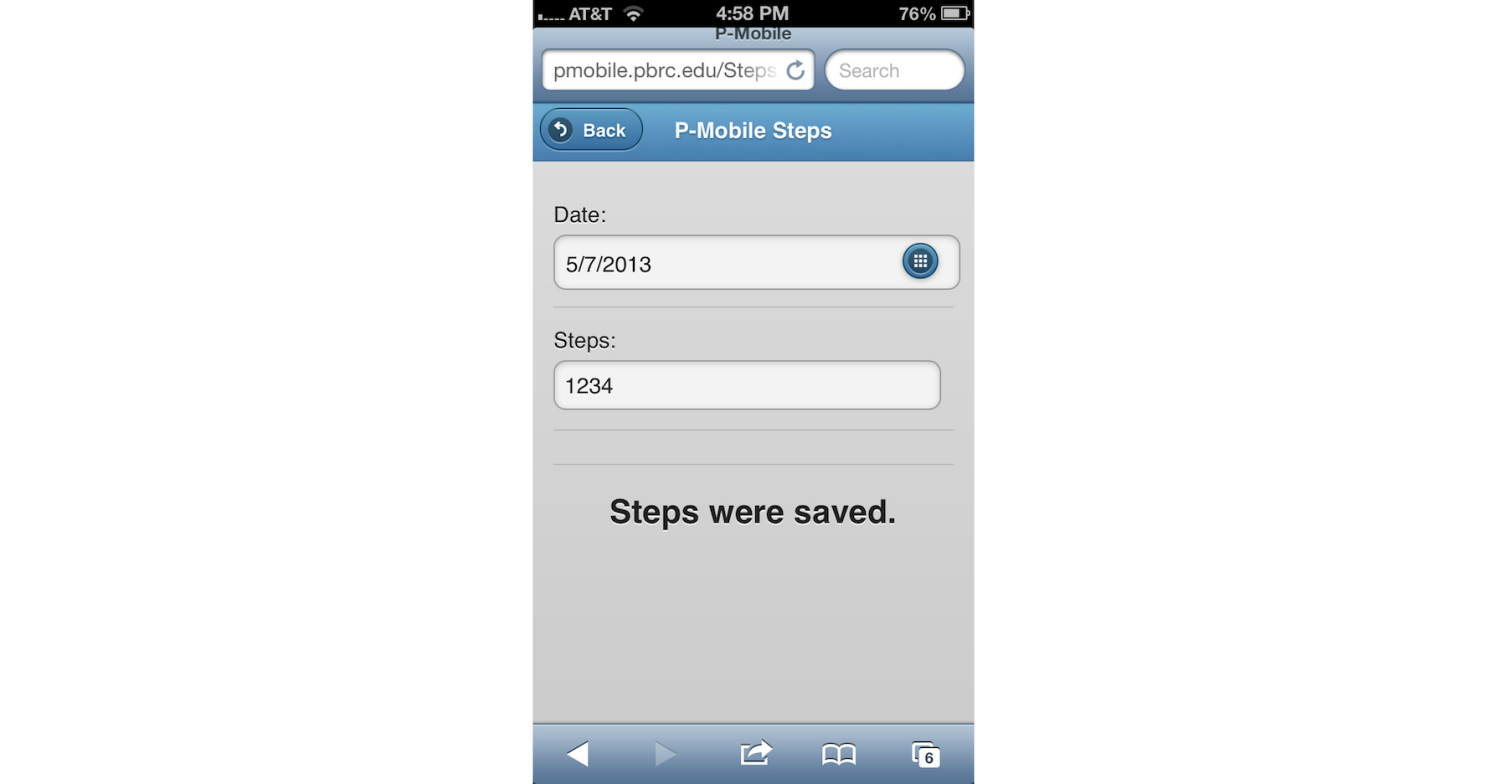
Mobile phone screenshot showing pedometer step count entry on P-Mobile website.

Parents in the MIG were given access to a version of the website (formatted for a mobile phone) in which they could view their child’s daily step goal, monitor their child’s step counts, and receive monthly nutrition tips (Table 1). The website provided parents with a target steps/day goal for their child, which was intended to increase their child’s physical activity by 1000, 3000, and 6000 steps/day above the child’s individualized baseline during the first, third, and fourth week of the intervention, respectively. The additional 6000 steps/day above the baseline level was to be maintained from weeks 4-12. This total increase of 6000 steps/day above baseline was selected to approximate the current national recommendation of 60 minutes of physical activity per day for children [40]. Parents in the MIG were instructed to use their mobile phone to access the study website to record their child’s step count each night after the child laid down to go to bed (Figure 2). Parents in the MIG were also sent monthly healthy nutrition tips via text message targeting the child in order to provide these families with potentially health promoting information.

**Table 1:** Components of the minimal (MIG) and intensive (IIG) intervention groups.

Intervention component	MIG	IIG
Access to mobile phone formatted website	X	X
6000 steps/day goal	X	X
Daily step monitoring	X	X
Monthly nutrition tips	X	
Weekly behavioral articles		X
Behavioral text messages		X
Steps/day graph		X

#### Intensive Intervention Group

**Figure 3:**
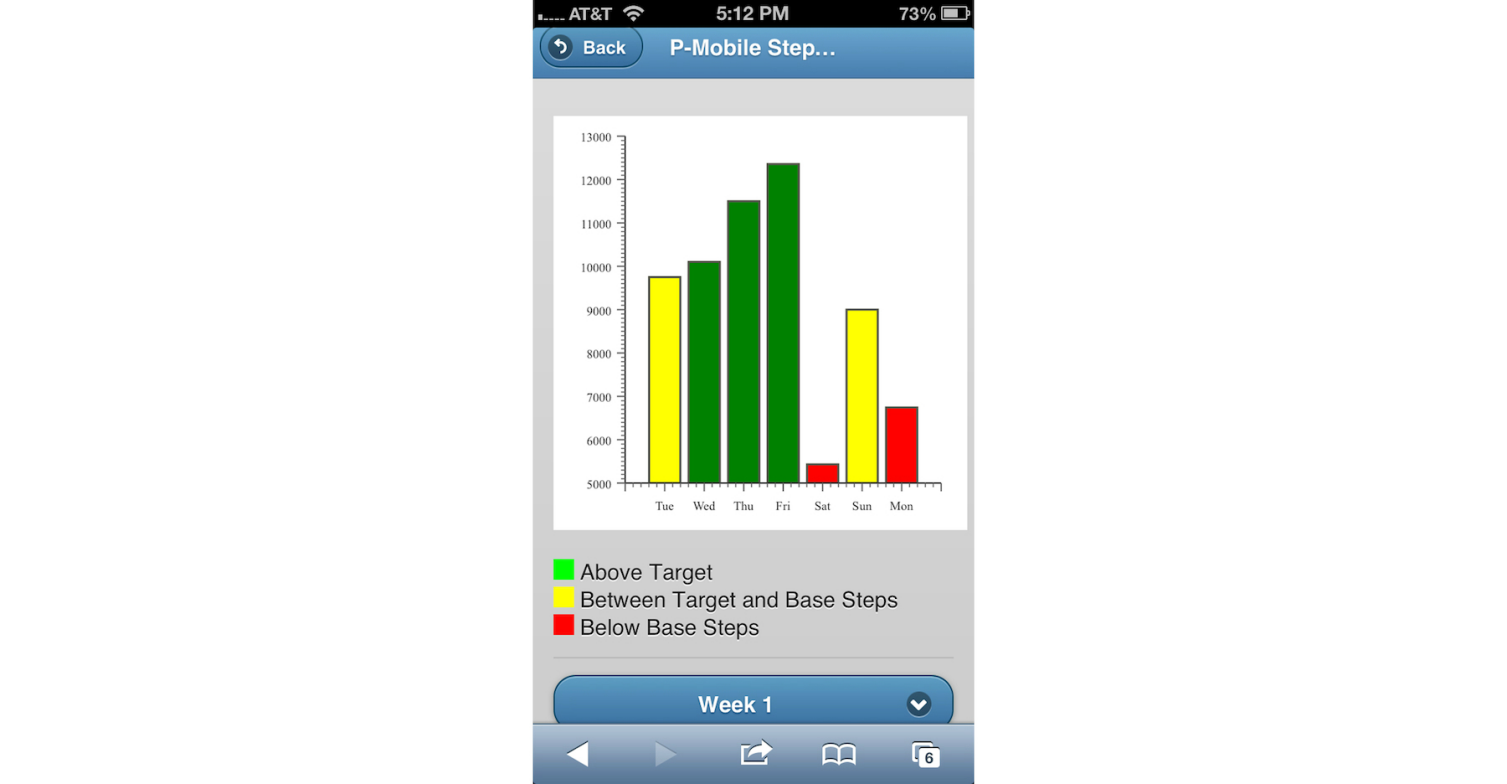
Mobile phone screenshot showing steps/day graph on P-Mobile website.

**Figure 4:**
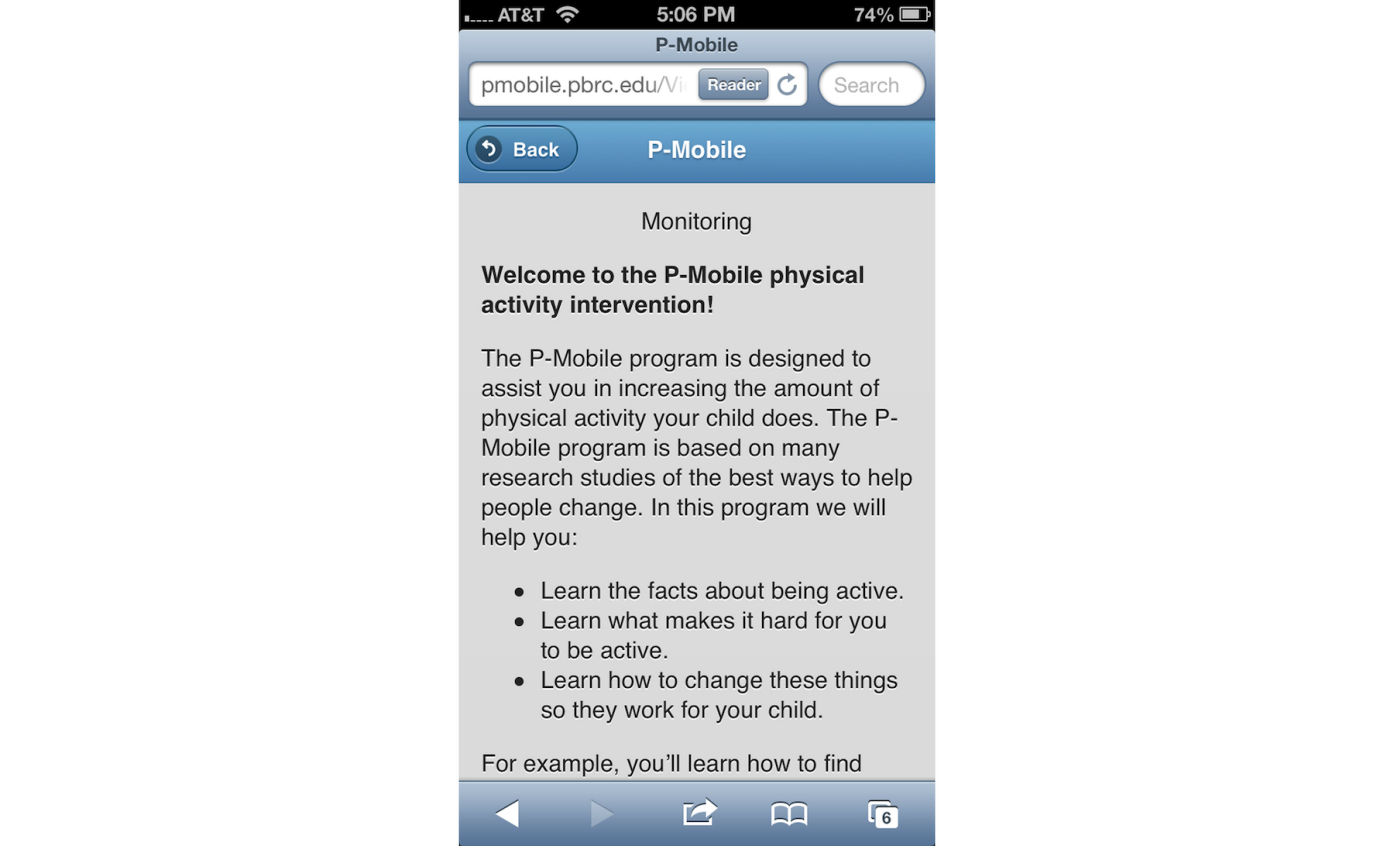
Mobile phone screenshot showing an article on P-Mobile website.

Parents in the IIG were given access to a version of the website in which they could view their child’s daily step goal, monitor their child’s step counts, view a steps/day graph, and read weekly behavioral articles, and they also received text messages. The step monitoring and steps/day website components and goals were identical to the MIG. The steps/day graph was color-coded to illustrate how their child’s daily steps compared to the target step goal: red bars represented days in which the child’s step count was below baseline, yellow represented step counts between baseline and the goal, and green represented step counts at or above the goal (Figure 3). Behavioral strategies based on the Social Cognitive Theory (Table 2) were adapted from previous interventions [41-43] and were delivered through weekly articles posted on the website (Figure 4) and via text messages. The average length of each behavioral article was 621 words. Each text message was ≤160 characters. Text messages were designed to prompt parents to encourage their child’s physical activity (eg, “This is a reminder for your child to be physically active!”), remind parents of behavioral concepts presented in the articles (article tip; eg, “A slip is a time where your child goes several days without reaching their activity goal. Try to stop slips as soon as you can.”), and motivate parents to foster behavioral change in their child (eg, “By engaging in regular physical activity, your child will reduce their risk of gaining weight.”).

**Table 2:** Behavioral articles provided to parents in the intensive intervention group (IIG).

Week	Title	Content
1	Self-monitoring	Parental monitoring of child activity, role of parent in child’s activity, increase steps/day by additional 1000.
2	Making time for exercise	Goal setting, scheduling time for activity, what is moderate intensity activity, maintain increased step/day of additional 1000.
3	Increasing activity outdoors	Benefits of outdoor play, role of the parents in child’s physical activity, increase steps/day by additional 2000.
4	Increasing activity indoors	Cues to activity, changing the home environment, increase steps/day by additional 3000 (achieve ≥ 6000 steps/day above baseline).
5	Checking-in #1	Identify barriers to achieving goal.
6	Problem solving	5-step problem-solving process.
7	Rewarding your child	Principles of positive reinforcement, rewards for increased activity.
8	Reducing sedentary time	Defining and identifying sedentary behavior, ways to reduce sedentary behaviors, substituting physical activity.
9	Checking-in #2	Identify barriers to achieving goal.
10	Lifestyle exercise	Incorporating activity that is part of daily living.
11	Parental modeling/social support	Parents as a role model for physical activity, obtaining social support from family members.
12	Relapse prevention	Defining and anticipating slips and relapses, ways to respond to slips.

### Measures

#### Overview

All measures, with the exception of the pedometers and the Home and Neighborhood Environment Questionnaire, were assessed at baseline and 12-weeks. The assessment staff was not blinded to the participant assignment.

#### Pedometer

Children in both groups were asked to wear a NL-1000 pedometer for the duration of the study. The device has a 7-day memory and steps are also digitally displayed on an immediately accessible screen. The NL-1000 contains the same internal mechanism as the NL-2000, which has been previously validated for counting steps in children [44]. Children were instructed to wear the pedometer during all waking hours except during water-based activities.

#### Anthropometrics

Height and weight were measured with the child dressed in normal street clothes, but without shoes and socks. Height was measured to the nearest 0.1 cm using a wall-mounted stadiometer (Holtain Ltd, Crymych, Dyfed, United Kingdom). Weight was measured to the nearest 0.1 kg using a digital scale (Indiana Scale Company model GSE 450). Waist circumference was measured to the nearest 0.1 cm at the natural waist, with clothing moved out of the way. The circumference was taken twice, with a third measurement taken if the first two were more than 0.5 cm apart. Body mass index (BMI) was calculated by dividing the participant’s average weight in kilograms by the square of their height in meters (kg/m^2^). BMI was converted to a *z*-score using gender and age data from the Centers for Disease Control and Prevention, to account for the fact that children of this age are still growing. Blood pressure and pulse were taken after the participant sat quietly for 5 minutes.

#### Body Composition

The Tanita Body Composition Analyzer (model TBF-240) was used to estimate body fat. The child stood on the scale with bare feet, and impedance data were recorded using a laptop computer. The Tanita has a mean difference of −1.0% with DXA and is considered to be very reliable in children [45].

#### Questionnaires

Children completed two questionnaires, with the assistance of a study staff member to ensure the child understood all questions. Parents were allowed to be present during the questionnaire administration if the child preferred. The 12-item Physical Activity Enjoyment Questionnaire (PACES) [46] was used to assess the level of a child’s enjoyment of various physical activities. The Child Depression Inventory-Short Form (CDI-S) [47] contains 10 items to assess symptoms of childhood depression and was used to measure self-reported depressed mood.

Parents completed three questionnaires. The Sedentary Behavior Questionnaire was completed to describe the amount of time their child spent watching TV, playing video games, playing on the computer, and doing other sedentary activities. The Home and Neighborhood Environment Questionnaire, adapted from the Neighborhood Impact on Kids study [48,49], assessed parent’s perceptions of their home and neighborhood environment, including safety, availability of destinations, and suitability of the neighborhood for walking and physical activity. The Food Frequency Questionnaire (FFQ) [50] was completed to describe their child’s food intake, including information about macro/micronutrients and food group servings.

### Statistical Analysis

Weekly steps/day means analyzed across all 12 weeks of the study were analyzed using a repeated measures analysis of variance. Change in the secondary outcome data (eg, BMI, body composition, questionnaires, website usage, etc) were analyzed using dependent samples *t* tests. Correlation coefficients were used to assess the relationships between secondary outcome measures and steps/day data. Effect sizes were calculated using Cohen’s *d*. All statistical analyses were conducted using SAS version 9.3.

## Results

### Baseline Characteristics

Characteristics of the participating children are summarized in Table 3. A total of 27 children (mean age 8.7, SD 1.4 years; 56%, 15/27, female; 59%, 16/27, African American) successfully completed the run-in period and were randomly assigned to the MIG (n=14) or IIG (n=13). The sample of 27 children had a mean BMI equal to 23.1 (SD 7.7) kg/m^2^, a mean BMI *z*-score equal to 1.5 (SD 1.0), a mean BMI percentile of 85.6 (SD 20.2) kg/m^2^, and mean waist circumference equal to 72.4 (SD 18.1) cm. Participating children across both intervention groups averaged 8621.8 steps/day and the difference in steps/day between the intervention groups was not statistically significant (MIG: 9042.5 vs IIG: 8168.6, *P*=.25). The only significant difference between intervention groups was percent of reported calories consumed from protein (MIG: 16.7% vs IIG: 19.1%; *P*=.047).

**Table 3:** Baseline demographic characteristics for all children.

Characteristic	All children (N=27)	Minimal intervention (n=14)	Intensive intervention (n=13)
	n (%) or mean (SD)	n (%) or mean (SD)	n (%) or mean (SD)
Age (years)	8.7 (1.4)	9.1 (1.3)	8.3 (1.5)
Gender (% female)	15/27 (56%)	7/14 (50%)	8/13 (62%)
Ethnicity (% African American)	16/27 (59%)	8/14 (57%)	8/13 (62%)
Height (cm)	138.1 (10.7)	140.0 (8.3)	135.9 (12.9)
Weight (kg)	45.5 (19.7)	44.5 (18.3)	46.5 (21.9)
BMI^a^ (kg/m^2^)	23.1 (7.7)	22.3 (7.4)	24.1 (8.1)
BMI *z*-score	1.5 (1.0)	1.3 (0.9)	1.7 (1.0)
BMI percentile	85.6 (20.2)	83.7 (21.0)	87.6 (20.1)
Waist circumference (cm)	72.4 (18.1)	70.5 (17.0)	74.5 (19.7)
Body fat percent	31.1 (11.2)	29.0 (10.3)	33.4 (12.1)
CDI-S^a^	47.1 (9.2)	47.3 (8.9)	46.8 (9.9)
Physical activity enjoyment	66.6 (7.0)	67.5 (7.9)	65.6 (6.2)
Sedentary time (weekday hours)	5.1 (4.0)	4.9 (4.1)	5.4 (3.9)
Sedentary time (weekend hours)	8.3 (5.4)	8.2 (5.0)	8.4 (5.9)
TV in room	16/27 (59%) yes	5/14 (36%) yes	11/13 (85%) yes
Total calorie consumption	1587.1 (647.5)	1520.6 (628.7)	1658.7 (685.1)
Steps/day	8621.8 (1955.0)	9042.5 (1930.5)	8168.6 (1953.2)

^a^BMI: body mass index

^b^CDI-S: Child Depression Inventory-Short Form

### Website Data

Parents across both groups logged into the website an average of 76.7 (SD 20.1) times over the course of the study (6.3 times/week). Parents were instructed to enter their child’s step counts daily, and parents in the MIG and IIG, respectively, entered 44.20% (520/1176) and 62.80% (686/1092) of their child’s step counts daily as instructed. Parents could also enter step count data for up to 7 days past the date the activity occurred. Another 40.60% (478/1176) and 27.00% (295/1092) of data was entered on a subsequent day for a total of 84.80% (997/1176) and 89.80% (981/1092) of step counts being entered by the parents in the MIG and IIG, respectively. The remaining 13.40% (158/1176; MIG) and 7.80% (85/1092; IIG) of the data were entered by the study coordinator because either the parents sent this information to the coordinator (via text message) or because the study coordinator contacted the parents (via phone) to retrieve missing data when identified. Overall, 97.90% (2220/2268; 98.20%, 1155/1176 for MIG; 97.60%, 1066/1092 for IIG) of expected step data were successfully entered by the parent or study coordinator.

**Figure 5:**
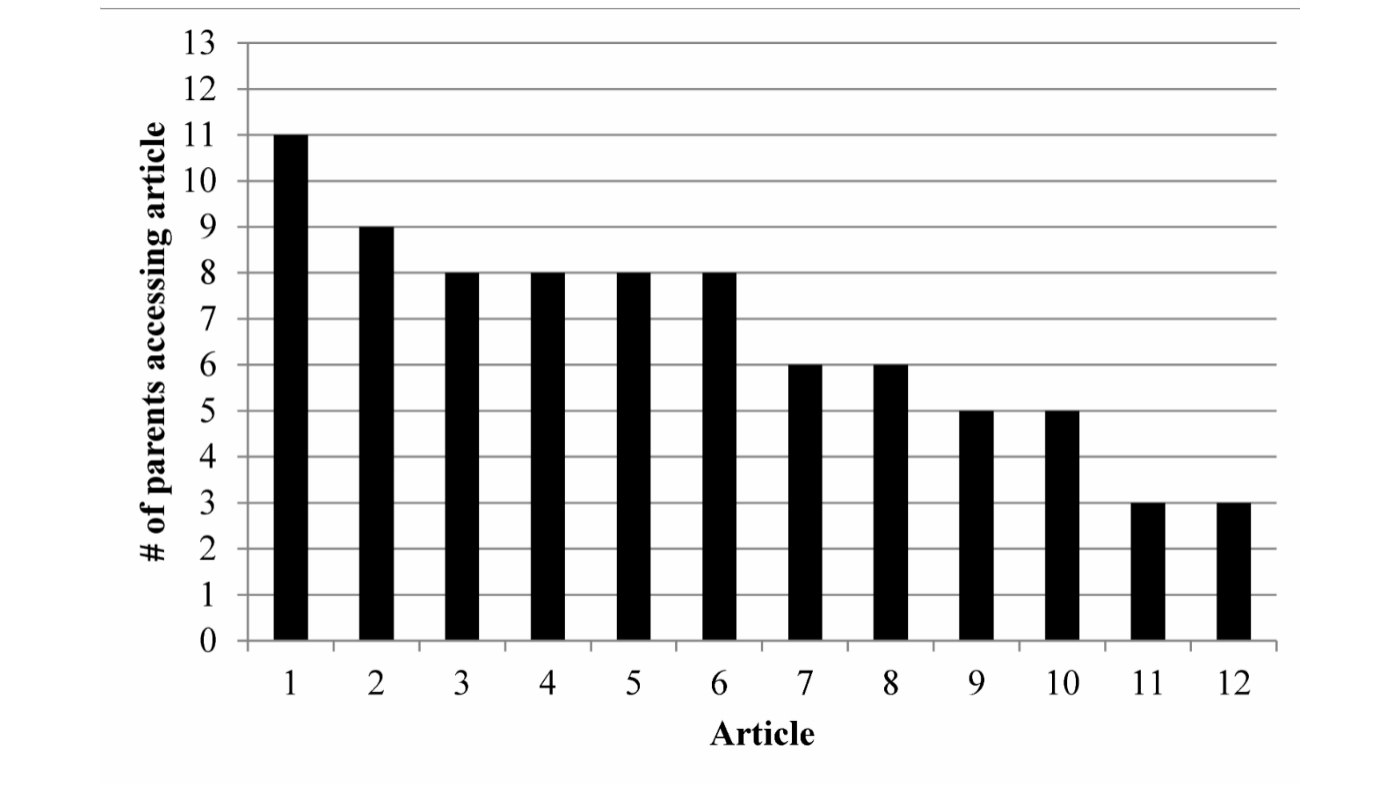
Number of parents in the Intensive Intervention Group (n=13) who accessed each of the 12 articles.

Figure 5 shows the number of parents in the IIG who accessed each of the weekly behavioral articles. Approximately 38% (10/27) of the parents accessed 9 or more articles, 23% (6/27) accessed between 4 and 8, and 38% (10/27) accessed less than 4 articles, with two parents never accessing an article. Overall, IIG parents accessed 70% (8/12) of the articles in Month 1, 60% (7/12) in Month 2, and 37.5% (5/12) in Month 3. Article accessing decreased significantly over the course of the study (*P*=.002).

Parents in the IIG visited the steps/day graph an average of 25.3 (SD 24.5) times over the course of the study (2.1 times/week). There was a clear dichotomy in access, with six participants accessing the graph fewer than 8 times, and seven accessing the graph more than 21 times.

### Text Messages

Parents in the MIG were sent 1-2 text messages and parents in the IIG were sent 7-8 text messages per week during the first 6 months of the study. However, daily text message reminders were implemented after the first four participants completed the study in an attempt to increase compliance with parent monitoring of the child’s daily steps. Therefore, parents in the MIG and IIG were sent approximately 7 and 13 text messages per week, respectively, averaged over the course of the study.

Parents in the MIG sent 162 (0.96/week) and parents in the IIG sent 419 (2.7/week) text messages over the course of the study. Approximately half of the text messages sent by parents in both groups were communications with the study coordinator regarding missing step data. The other text messages sent by the parents were related to equipment/resource issues (eg, pedometer, website), requests for further information (eg, spontaneous questions, scheduling), or responses to a text message they had received.

### Step Counts

**Figure 6:**
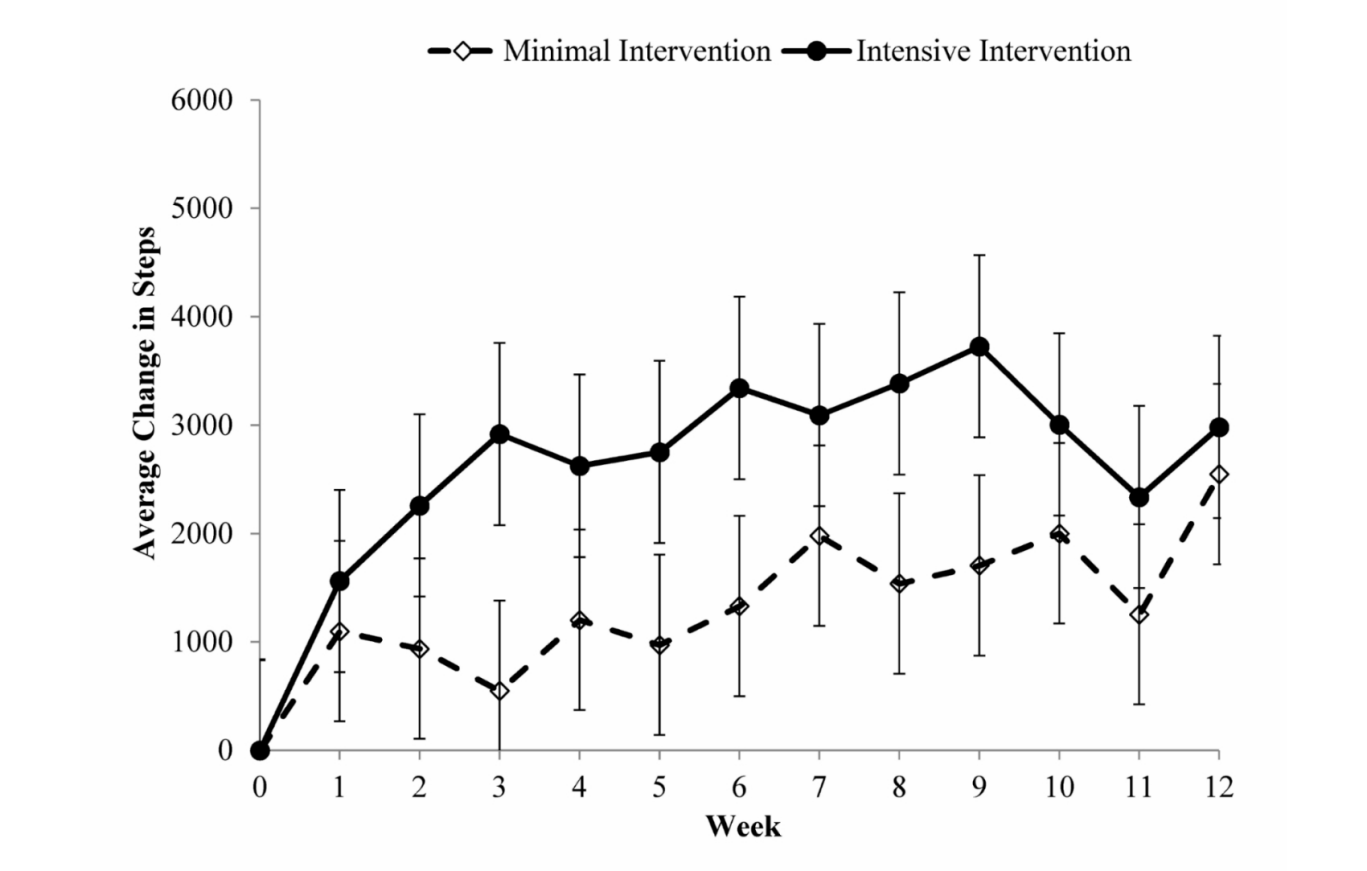
Average change in child’s steps per week by intervention group.

All randomized children attended the Week 12 visit and thus completed the 12-week study. Figure 6 graphically illustrates the weekly changes in steps/day for the two intervention groups. Children in the MIG and IIG both demonstrated significant increases across the 12 weeks by 1427.6 (SD 583.0; *P*=.02) and 2832.8 (SD 604.9) steps/day (*P*<.001) above baseline, respectively. The between-group difference was not statistically significant (*P*=.10) yet the effect size was *d*=.40. The group by time interaction was not significant (*P*=.57).

### Secondary Analyses

Table 4 shows that none of the changes in body composition variables, including BMI, BMI *z*, waist circumference, body fat percent, and fat free mass were significant (all *P* values >.22). Further, there were no significant within or between-group differences in the parent proxy-reported measures, including sedentary behavior and food intake, or child self-reported physical activity enjoyment, and depressive symptoms (all *P* values >.38). Therefore, the data from the groups were combined to assess the relationship between the change in these secondary outcome measures and change in steps/day. The correlation between change in physical activity enjoyment and change in steps/day was statistically significant (*r*=.469; *P*=.003). For participants in the IIG, a significant correlation (*r*=.575, *P*=.04) was observed between the number of articles accessed by the parent and average change in their child’s steps/day.

**Table 4:** Change in outcome variables from baseline to 12 weeks.

	Minimal intervention	Intensive intervention	*P* value (between group differences)
	mean (SD)	mean (SD)	
Height (cm)	1.5 (1.0)	1.6 (1.1)	.843
Weight (kg)	0.94 (2.1)	1.4 (1.7)	.536
BMI^a^ (kg/m^2^)	−0.86 (1.1)	0.28 (1.0)	.369
BMI *z*-score	0.016 (0.19)	0.00 (0.14)	.773
BMI percentile	1.01 (6.1)	0.27 (1.9)	.455
Waist circumference (cm)	0.85 (4.0)	1.3 (2.8)	.764
Body fat percent	−0.24 (1.9)	0.69 (2.4)	.275
CDI-S^b^	−1.1 (4.0)	−3.4 (8.4)	.378
Physical activity enjoyment	−0.09 (0.17)	−0.01 (0.29)	.391
Sedentary time (weekday hours)	0.0 (3.2)	−0.59 (3.9)	.617
Sedentary time (weekend hours)	−1.2 (4.0)	−1.1 (5.5)	.941
Total calorie consumption	96.2 (682.4)	−310.6 (569.1)	.200
% calories from fat	−1.8 (6.7)	−0.06 (7.2)	.483
% calories from protein	0.78 (2.6)	0.59 (2.3)	.976
% calories from carb	1.0 (5.7)	−0.37 (7.2)	.323
Steps/day^c^, mean (SE)	1427.6 (583.0)	2832.8 (604.9)	.102

^a^BMI: body mass index

^b^CDI-S: Child Depression Inventory-Short Form

^c^Represents the average change in weekly steps/day across 12 weeks

## Discussion

### Principal Results

The P-Mobile study demonstrated that it is feasible to deliver a child-targeted physical activity promotion program to parents through their mobile phones. Feasibility was demonstrated by parental utilization of the components of the intervention, including entering their child’s step counts, responding to text messages, and accessing the behavioral articles. The intervention also resulted in increased physical activity in both study groups. Step counts increased significantly in both the MIG and the IIG over the course of the 12-week intervention. These findings suggest that mobile phone-based physical activity promotion interventions delivered to parents have the potential to be utilized and may positively affect physical activity levels in children.

Parental use of the intervention components varied by the component assessed. Concerning step count data, only half of the parents complied with the study requirement to enter step counts each night. This required the study coordinator to prompt parents for about half of the data and enter 10%-15% of the data. Therefore, the large volume of step data entered was the result of combined efforts by both the parents and the study coordinator, which may be difficult for participants and burdensome on study staff to sustain over a period longer than 12 weeks. Bluetooth capable activity monitors (eg, FitBits, Jawbone, Garmin Vivofit, etc) may lessen this burden and increase compliance. Concerning text messages, on average, parents in the IIG received approximately 13 automated text messages per week. This level appears to be tolerable because only one parent (4%, 1/27) requested a decrease in the frequency of text messages. Parents sent between 1 and 3 text messages per week to the study coordinator, but this was largely related to obtaining missing step data. Text messaging appears to be an acceptable form of communication, but it did not appear to be utilized by parents to increase their child’s physical activity levels. Concerning articles, accessing article content was positively associated with change in steps/day for families in the IIG. However, accessing article content decreased significantly over the course of the study. This finding is consistent with Internet-based studies reporting incrementally reduced usage of websites across the study duration [51,52] and suggests that this type of intervention may not be ideally suited for all parents. In sum, although the components of the intervention were utilized, they were not utilized as anticipated. Based on our results, future studies should find ways to maintain consistent engagement of participants in mobile phone-based interventions because performance is positively associated with engagement.

The P-Mobile study demonstrated that children in both the MIG and IIG significantly increased steps/day above baseline levels. The intervention where parents received additional behavioral strategies and text messages (IIG) resulted in steps/day increases that were two-fold greater than steps/day levels reported with only daily monitoring and goal setting (MIG). Though this difference was not statistically significant, suggestion of a moderate treatment effect was observed. However, the children in P-Mobile were unable to achieve the study goal (increasing 6000 steps/day above individualized baseline levels) directed to their parents. Children in the MIG were able to reach approximately 24%, while children in the IIG were able to reach approximately 50% of this goal. A 6000 steps/day increase from baseline levels would have amounted to ~13,500 steps/day for girls and ~16,000 steps/day for boys. Surveillance studies of free-living behavior demonstrate that only 25% of girls and 15% of boys aged 6-10 years achieve this level of habitual daily physical activity [53]. Therefore, the goals promoted herein were achievable, but require intervention support for those not habitually inclined toward a physically active lifestyle. Given the results of the current study combined with those of previous investigations, it suggests that interventions need to be further strengthened in order for sedentary children to achieve this level of activity. Interventions can potentially be further strengthened by engaging both parents (eg, providing behavior strategies to both parents, encouraging both parents to exercise with their child), incorporating siblings into the intervention (eg, delivering the intervention to all children in the family), and/or gathering real-time data (eg, through ecological momentary assessment) to better understand the physical activity patterns to determine optimal times/ways to incorporate physical activity in each individual child’s life [54]. A potential downside to such strengthening efforts is the possible increase in staff and participant burden, and the potential for increased non-compliance due to the more intense strategies and requirements. Further research will help illuminate the optimal approach to using this technology to promote children’s physical activity by targeting parents. For example, a multiphase optimization strategy [55,56] can be used to develop the ideal approach from the many intervention components (eg, pedometers, text messages, lesson plans, etc) of P-Mobile.

### Comparison to Prior Work

Family-based studies that have used pedometers to increase physical activity in children have typically delivered the intervention through group sessions [57-60]. A consistent finding across these interventions is that they realize increases in children’s physical activity [15], anywhere from ~1500 [59,60] to ~3000 [57] steps/day above baseline levels. One study showed a significant differential increase of ~1000 steps between the intervention and control groups [58]. In two studies that utilized mobile phones to deliver behavioral change strategies and pedometers as monitoring tools to increase physical activity in children [24,29], neither resulted in significant within or between-group differences in steps/day. P-Mobile showed increases in physical activity for children in both groups (~1400 to ~2800 steps/day) and resulted in differences between groups (~1400 steps/day) that are within the range of values of similar family-based interventions that delivered the intervention through face-to-face contact. To our knowledge, P-Mobile is the only mobile phone-delivered physical activity intervention in children that has resulted in significant within group increases in steps/day. Therefore, it appears that mobile phone interventions can be delivered to parents and result in increases in physical activity in their children. Our findings are in need of replication with larger samples, over longer durations, and with more diverse populations.

### Limitations

The P-Mobile findings should be interpreted within the context of the study’s limitations. One major limitation was the small sample size. This may have provided insufficient power for detecting statistical significance between the observed group differences. The study had 17% power to detect differences and would have needed a sample size of 105 children/group to detect a 1400 steps/day difference. In addition, the study was limited to only 12 weeks; therefore, the long-term effectiveness of the study is unknown. The mobile phone components utilized in this study were limited to text messages and accessing a website through the mobile phone. Smartphones offer increased functionality, such as specially designed apps that could have fostered automated uploading of step data, thereby reducing participant burden and potentially increasing utilization. Apps also allow for location-based services that can be used to assist participants in identifying nearby facilities conducive for physical activity. Concerning measurement, although we used an objective measure of activity, pedometers do not capture all activity such as biking and swimming, and dietary intake was assessed using the FFQ, which is known to provide biased estimates compared to gold standard techniques, such as doubly labeled water. Finally, P-Mobile did not contain a control group. Our comparison group, the MIG, increased their average steps/day across the study, while control groups in previous studies have not significantly increased steps/day or have resulted in decreased steps/day [61,62].

### Conclusions

P-Mobile was able to demonstrate that it is both feasible and effective to deliver a physical activity promotion program utilizing mobile phones. The study also showed that parents can be the exclusive targets and thus agents of their children’s behavior change. Our findings add to the literature indicating that pedometers can be used to help increase physical activity in children. Our program relied on text messages, a website, involved few families, and was of a moderate duration. Researchers can build upon this foundation to develop more effective mobile phone-based interventions targeting childhood physical activity.

## Abbreviations

BMI: body mass index
CDI-S: Child Depression Inventory-Short Form
CVD: cardiovascular disease
FFQ: Food Frequency Questionnaire
IIG: intensive intervention group
MIG: minimal intervention group
NL-1000: New Lifestyles 1000 pedometer
PACES: Physical Activity Enjoyment Questionnaire
P-Mobile: Parent-Targeted Mobile Phone Intervention to Increase Physical Activity in Sedentary Children
TBF: Tanita Body Composition Analyzer
